# Books Review

**Published:** 1871-01

**Authors:** 


### Books Review.

*Bumstead on Venereal Diseases.* Third Edition, Revised and Enlarged, with Illustrations. H. C. LEA, Philadelphia. 1870.

This work on Venereal Diseases has already found its way into the library of almost all American physicians, who have any desire to conform their views and practices in this class of diseases to the teachings of recent observation.

This edition has been enlarged and improved, yet contains essentially the same text of former editions. and, as now presented to the American profession, will everywhere constitute its standard.

The questions which have divided professional opinion are being rapidly solved, and truth is being attained, which will commend itself to all. Indeed, venereal diseases, as now understood and taught, are settled upon the basis of easily demonstrated truth. No work in this country has done so much towards correcting professional error in venereal diseases as this one of Prof. Bumstead, who has presented truth in such forcible manner as to carry conviction. It is to be hoped that all physicians who have not already done so, will study venereal diseases anew, accepting the light which modern experience and observation has thrown upon the subject. No author can be more heartily commended, or is more worthy as a guide, both in the pathology and in the treatment of venereal diseases.

---

*Practical Anatomy: A Manual of Dissections.* By C. HEATH, F. R. C. S. Edited by WM. W. KEEN, M. D. HENRY C. LEA. Philadelphia, 1871.

It appears to us certain that, as a guide in dissection, and as a work containing the facts of anatomy in brief, and easily understood form, this manual is complete. This work contains, also, very perfect illustrations of parts which can thus be more easily understood and studied; in this respect it compares favorably with works of much greater pretention. Such manuals of Anatomy are always favorite works with medical students. We would earnestly recommend this one to their attention: it has excellencies which make it valuable as a guide in dissecting, as well as in studying anatomy.

---

*Galvano Therapeutics.* The Physiological and Therapeutical Action of the Galvanic Current upon the Acoustic, Optic, Sympathetic and Pneumogastric Nerves. By WILLIAM B. NEFTEL, M. D. D. APPLETON & CO., New York, 1871.

Those of our readers curious in this matter, will be deeply interested in this little work before us. It possesses merits in many respects; the chief one to medical practitioners is its brevity. It contains, in a very small compass, much of the present views in Galvano Therapeutics; and physicians who desire to test the value of this agent in the treatment of these forms of nervous diseases, will find all the instruction that the present state of medical knowledge upon this subject will permit.

